# Bornyl *cis*-4-Hydroxycinnamate Suppresses Cell Metastasis of Melanoma through FAK/PI3K/Akt/mTOR and MAPK Signaling Pathways and Inhibition of the Epithelial-to-Mesenchymal Transition

**DOI:** 10.3390/ijms19082152

**Published:** 2018-07-24

**Authors:** Tzu-Yen Yang, Mei-Li Wu, Chi-I Chang, Chih-I Liu, Te-Chih Cheng, Yu-Jen Wu

**Affiliations:** 1 Department of Food Science, National Pingtung University of Science and Technology, Pingtung 91201, Taiwan; gini0307@yahoo.com.tw (T.-Y.Y.); mlwu@mail.npust.edu.tw (M.-L.W.); 2 Department of Biological Science and Technology, National Pingtung University of Science and Technology, Pingtung 91201, Taiwan; changchii@mail.npust.edu.tw; 3 Department of Nursing, Mei-ho University, Pingtung 91202, Taiwan; x00002177@meiho.edu.tw (C.-I.L.); x00003077@meiho.edu.tw (T.-C.C.); 4 Department of Biological Technology, Meiho University, Pingtung 91202, Taiwan; 5 Department of Beauty Science, Meiho University, Pingtung 91202, Taiwan

**Keywords:** cell invasion, cell migration, melanoma, *Piper betle* stems, bornyl *cis*-4-hydroxycinnamate, epithelial to mesenchymal transition

## Abstract

Bornyl *cis*-4-hydroxycinnamate, a bioactive compound isolated from *Piper betle* stems, has the potential for use as an anti-cancer agent. This study investigated the effects of bornyl *cis*-4-hydroxycinnamate on cell migration and invasion in melanoma cells. Cell migration and invasion were compared in A2058 and A375 melanoma cell lines treated with/without bornyl *cis*-4-hydroxycinnamate (1–6 µM). To examine whether bornyl *cis*-4-hydroxycinnamate has a potential anti-metastatic effect on melanoma cells, cell migration and invasion assays were performed using a Boyden chamber assay and a transwell chamber in A2058 and A375 cells. Gelatin zymography was employed to determine the enzyme activities of MMP-2 and MMP-9. Cell lysates were collected for Western blotting analysis of matrix metalloproteinase (MMP)-2, MMP-9 and tissue inhibitors of metalloproteinase-1/2 (TIMP-1/2), as well as key molecules in the mitogen-activated protein kinase (MAPK), focal adhesion kinase (FAK)/ phosphatidylinositide-3 kinases (PI3K)/Akt/ mammalian target of rapamycin (mTOR), growth factor receptor-bound protein 2 (GRB2) signaling pathways. Our results demonstrated that bornyl *cis*-4-hydroxycinnamate is a potentially useful agent that inhibits melanoma cell migration and invasion, and altered melanoma cell metastasis by reducing MMP-2 and MMP-9 expression through inhibition of the FAK/PI3K/Akt/mTOR, MAPK, and GRB2 signaling pathways. Moreover, bornyl *cis*-4-hydroxycinnamate inhibited the process of the epithelial-to-mesenchymal transition in A2058 and A375 melanoma cells. These findings suggested that bornyl *cis*-4-hydroxycinnamate has potential as a chemotherapeutic agent, and warrants further investigation for its use in the management of human melanoma.

## 1. Introduction

Melanoma, a malignancy of melanocytes found predominantly in the skin, is the most dangerous form of cutaneous cancer, and its incidence is increasing throughout the world [1]. Surgery is the main treatment for early-stage melanoma [2,3], but it is rarely curative for the advanced stages. The therapy for most patients with advanced stages melanoma is chemotherapy and cytokine therapy; however, it presents poor responses. Dacarbazine, an anti-neoplastic agent used against malignant melanoma, has a response rate of 15–25% [4,5]. High-doses of interleukin (IL)-2, a form of cytokine therapy, has a response rate of only around 12.5% [6,7]. On the other hand, patients may receive complex targeted therapy or immunotherapy. Although some patients initially responded to treatment, they later develop resistance problems. Indeed, metastatic melanoma has a very poor prognosis, with a low survival rate [8,9,10]. Therefore, development of new drugs to treat patients with malignant melanoma is an important issue.

Bornyl *cis*-4-hydroxycinnamate is an active compound isolated from *Piper betle* stems. *Piper betle* Linn, belonging to the Piperaceae family, is an evergreen and perennial vine with heart-shaped leaves with a glossy surface. *Piper betle* is generally found in hot and moist areas, such as Southeast Asia, Malaysia, Guangdong, Java, Indonesia, the Philippine Islands, East Africa, and Taiwan. In Taiwan, it is found in Nantou, Pingtung, Taitung, Green Island, and Orchid Island (Lanyu). *Piper* species have been used in a variety of traditional medicines, including traditional Chinese medicine, folklore medicine of Latin America, and the Ayurvedic therapies of the West Indies. *Piper betle* contains important chemical constituents such as allylpyrocatechol diacetate, allylpyrocatechol monoacetate, campene, caryophyllene, chavibetol, chavibetol acetate, chavibetol methyl ether, 1-8-cineol, eugenol, u-limonene, a-pinene, f-pinene, and saprobe. These components are valued as stimulants of antibacterial [11], antileishmanicidal [12], antifilarial [13], anti-fungal [14], antimalarial [15], larvicidal [16], and anti-proliferative [17] activities, and details of their activities have been reviewed by Rekha el al. [18] and Bhuvaneswari et al. [19]. Furthermore, *Piper betle* extract has also been found to have hepatoprotective and anti-cancer properties [20,21,22,23,24]. Recently, bornyl *cis*-4-hydroxycinnamate has been shown to inducing apoptosis in melanoma cells. [25]. In order to evaluate the anti-cancer cell migration and invasion effects of *Piper betle* against melanoma, we examined the effect of bornyl *cis*-4-hydroxycinnamate on human melanoma cell lines A2058 and A375 in the present study. The current results suggested that bornyl *cis*-4-hydroxycinnamate may be a potential therapeutic agent for human melanoma treatment.

## 2. Results

### 2.1. The Cytotoxic Effects of Bornyl cis-4-Hydroxycinnamate on A2058 and A375 Cells

**Figure 1:**
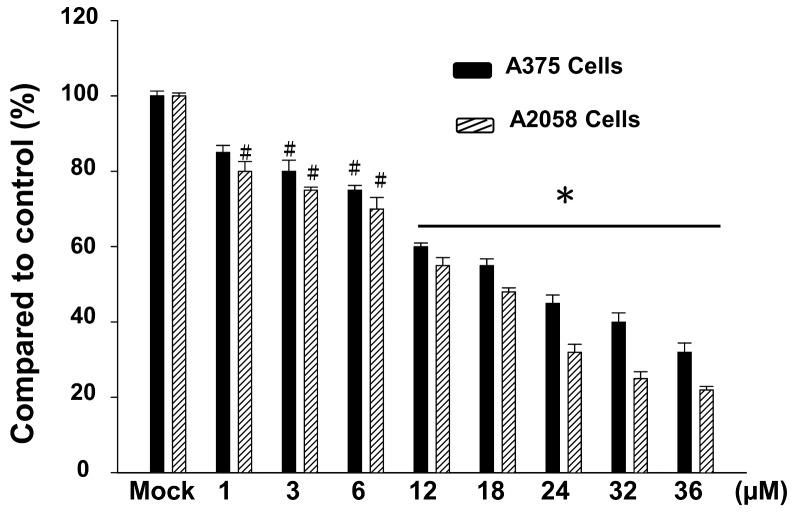
Cell viabilities of A2058 and A375 cells after bornyl *cis*-4-hydroxycinnamate treatment and vehicle control (dimethyl sulfoxide; DMSO) treatment for 24 h. A methylthiazole tetrazolium (MTT) assay showed the cytotoxic effects of bornyl *cis*-4-hydroxycinnamate treatment on A2058 and A375 cells in a concentration-dependent manner (* *p* < 0.001, # *p* < 0.05). The results were obtained from three independent experiments.

The cytotoxic effects of bornyl *cis*-4-hydroxycinnamate on human melanoma cell lines A2058 and A375 were quantitated using a methylthiazole tetrazolium (MTT) assay. Figure 1 shows the cytotoxic effects of bornyl *cis*-4-hydroxycinnamate at different concentrations (0, 1, 3, 6, 12, 18, 24, 32, and 36 μM) on A2058 and A375 melanoma cells. The results revealed that bornyl *cis*-4-hydroxycinnamate inhibited the cell viability of A2058 and A375 melanoma cells in a dose-dependent manner (* *p* < 0.001, # *p* < 0.05) (Figure 1). At a concentration of 12 μM, bornyl *cis*-4-hydroxycinnamate significantly inhibited the cell viability of A2058 and A375 cells (Figure 1). We also evaluated the viability of another melanoma cell lines (B16–F10 cells) within various concentrations of bornyl *cis*-4-hydroxycinnamate by MTT assay. The results displayed that the viability of B16–F10 cells were similar to A2058 cells and A375 cells (Supplementary Figure S1).We chose a concentration (1, 3, and 6 μM) of bornyl *cis*-4-hydroxycinnamate for all subsequent experiments. To ensure that the inhibitory effects of bornyl *cis*-4-hydroxycinnamate on A2058 and A375 cells migration and invasion were not caused by the cytotoxicity of the compound, we selected fibroblast cells (WS-1 cells) for cell cytotoxicity analysis. The results showed that the bornyl *cis*-4-hydroxycinnamate had less cell cytotoxicity compared to WS-1 cells (Supplementary Figure S2).

### 2.2. Bornyl cis-4-Hydroxycinnamate Inhibited Migration and Invasion of A2058 and A375 Cells

**Figure 2:**
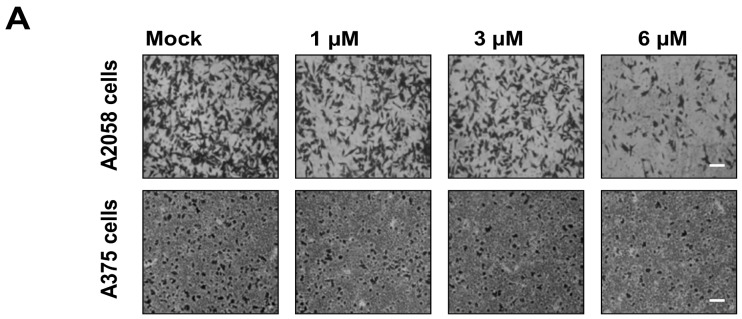

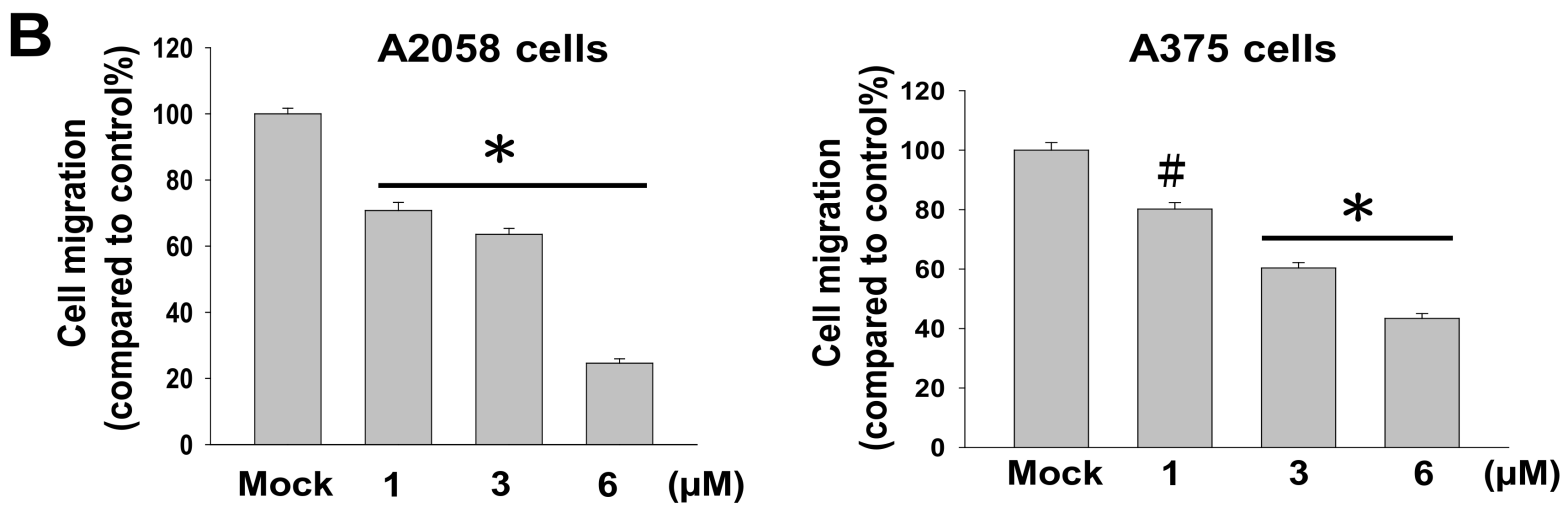
The effects of bornyl *cis*-4-hydroxycinnamate on A2058 and A375 cell migration. (**A**) After 24 h of treatment with bornyl *cis*-4-hydroxycinnamate, the percentages of migrated A2058 and A375 cells were significantly reduced in comparison with the controls (Mock: cells treated with vehicle DMSO). Scale bars = 20 μm. The results shown here are representative of three independent experiments; (**B**) Quantitative analysis of three independent experiments showed that bornyl *cis*-4-hydroxycinnamate suppressed A2058 and A375 cell migration in a dose-dependent manner (# *p* < 0.05, * *p* < 0.001 as compared with controls). Results were from three independent experiments in triplicate in each experiment.

**Figure 3:**
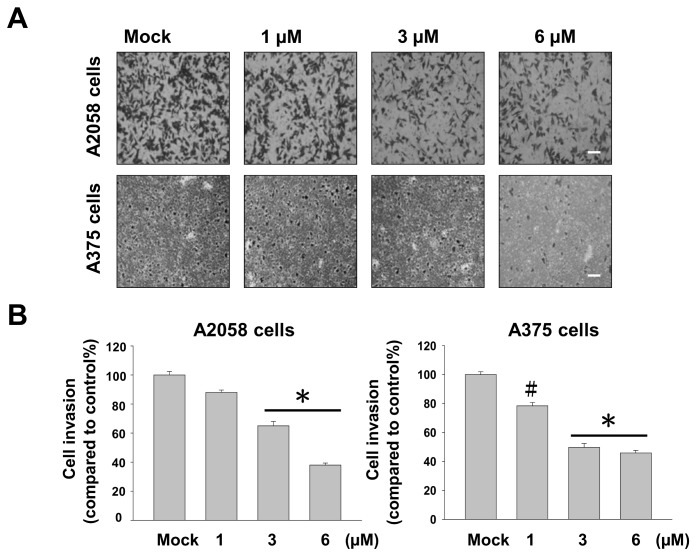
Inhibition effect of bornyl *cis*-4-hydroxycinnamate on A2058 and A375 cell invasion through Matrigel-coated transwells. (**A**) Bornyl *cis*-4-hydroxycinnamate significantly reduced the numbers of A2058 and A375 cells that invaded through the membrane as compared with the controls. The results shown here are representative of three independent experiments. Mock: cells treated with vehicle control (DMSO). Scale bars = 20 μm; (**B**) Quantitative analysis of three independent experiments showed that bornyl *cis*-4-hydroxycinnamate suppressed A2058 and A375 cell invasion in a dose-dependent manner (# *p* < 0.05, * *p* < 0.001 as compared with controls).

Cell–matrix interaction and cell motility are two major factors that determine the metastatic properties of cancer cells. To examine whether bornyl *cis*-4-hydroxycinnamate has a potential anti-metastatic effect on melanoma cells, cell migration and invasion assays were performed using a Boyden chamber assay and a transwell chamber, respectively, in A2058 and A375 cells. The results indicated that cell migration of A2058 and A375 cells was significantly inhibited by bornyl *cis*-4-hydroxycinnamate in a dose-dependent manner. Moreover, the migratory abilities of A2058 and A375 cells were reduced to approximately 70% and 50%, respectively, after treatment with 6 μM bornyl *cis*-4-hydroxycinnamate for 24 h (Figure 2), and their invasive abilities were reduced by about 50% in both cell lines (Figure 3). These results suggested that bornyl *cis*-4-hydroxycinnamate inhibits melanoma cell migration and invasion.

### 2.3. Bornyl cis-4-Hydroxycinnamate Reduced the MMP-2/-9 Activities and Regulated the Expressions of MMP-2, MMP-9, uPA, TIMP-1, and TIMP-2 Proteins in A2058 and A375 Cells

**Figure 4:**
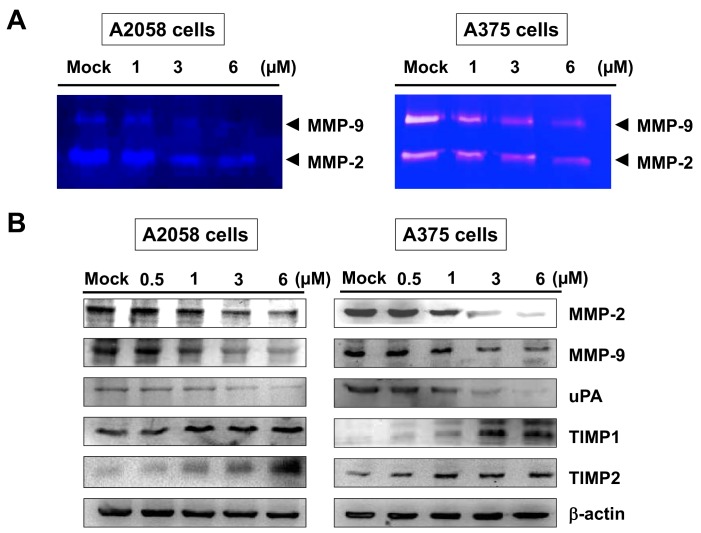
Effects of bornyl *cis*-4-hydroxycinnamate on the activities of MMP-2/-9 and protein levels in A2058 and A375 cells. A2058 and A375 cells were treated with different concentrations of bornyl *cis*-4-hydroxycinnamate (0, 1, 3, 6 µM) for 24 h, and conditioned media and cell lysates were collected for analysis. (**A**) Gelatin zymography showed that bornyl *cis*-4-hydroxycinnamate inhibited MMP-2/-9 activities in A2058 and A375 cells in a dose-dependent manner; (**B**) The expression levels of MMP-2/-9-related proteins, including MMP-2, MMP-9, uPA, TIMP-1, and TIMP-2, were validated by western blotting. In order to observe the effect of these proteins associated with bornyl *cis*-4-hydroxycinnamate at low concentrations, a 0.5 μM treated group was added. The results showed decreased MMP-2/-9 and uPA protein levels and increased TIMP-1 and TIMP-2 protein levels in A2058 and A375 cells after bornyl *cis*-4-hydroxycinnamate treatment. β-actin was used as the protein loading control. Mock: cells treated with vehicle control (DMSO).

Gelatin zymography was employed to determine the enzyme activities of MMP-2 and MMP-9 in the invasion of A2058 and A375 cells. A2058 and A375 cells were maintained in serum-free media with bornyl *cis*-4-hydroxycinnamate (0, 1, 3, 6 µM) for 24 h, and the activities of MMP-2 and MMP-9 in the culture conditioned medium were measured by gelatin zymography. The activities of MMP-2 and MMP-9 were reduced by bornyl *cis*-4-hydroxycinnamate treatment in a dose-dependent manner, as shown in Figure 4A. In order to understand whether specific endogenous tissue inhibitors of metalloproteinases (TIMPs) and serine protease urokinase plasminogen activator (uPA) contribute to regulate the activities of MMP-2 and MMP-9 in terms of the invasiveness, metastasis, and prognosis of melanoma, we further determined the effects of bornyl *cis*-4-hydroxycinnamate on the regulation of the expressions of MMP-2, MMP-9, uPA, TIMP-1, and TIMP-2 proteins by Western blotting assay. The protein levels of MMP-2, MMP-9, and uPA were decreased, and those of TIMP-1 and TIMP-2 were increased, in A2058 and A375 cells after treatment with bornyl *cis*-4-hydroxycinnamate for 24 h (Figure 4B).

### 2.4. Bornyl cis-4-Hydroxycinnamate Inhibited FAK/PI3K/Akt/mTOR Signaling Pathway-Associated Proteins

**Figure 5:**
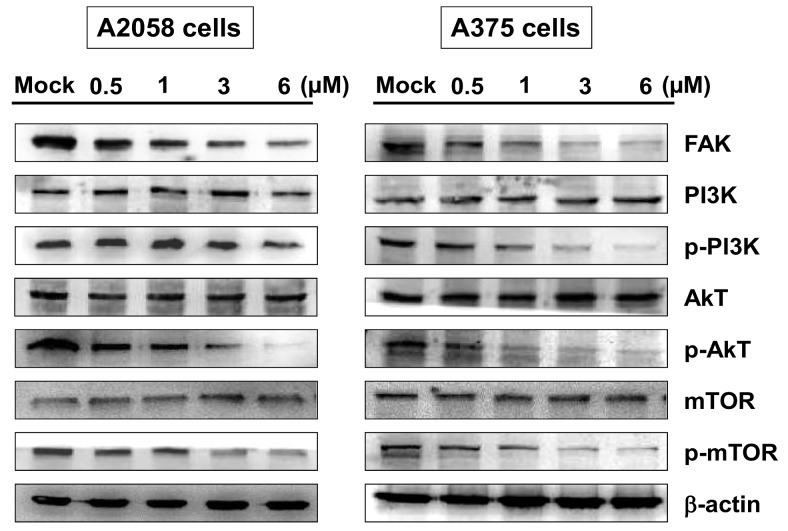
The effects of bornyl *cis*-4-hydroxycinnamate on the FAK/PI3K/Akt/mTOR signaling pathways in A2058 and A375 cells. A2058 and A375 cells were treated with bornyl *cis*-4-hydroxycinnamate at various concentrations (0, 0.5, 1, 3, 6 µM) for 24 h, and cell lysates were collected for Western blot analysis. The FAK/PI3K/Akt/mTOR-related proteins were validated, including FAK, PI3K, p-PI3K, Akt, p-Akt, mTOR, and p-mTOR. Bornyl *cis*-4-hydroxycinnamate inhibited FAK, p-PI3K, p-Akt, and p-mTOR protein levels in A2058 and A375 cells. β-actin was used as the protein loading control. Mock: cells treated with vehicle control (DMSO).

Overexpression and activation of focal adhesion kinase (FAK) are known to be associated with cancer metastasis. To determine the activity of FAK and downstream signaling pathways in A2058 and A375 cells upon bornyl *cis*-4-hydroxycinnamate treatment, FAK/PI3K/Akt/mTOR signaling-associated proteins, including Akt, p-Akt, PI3K, p-PI3K, mTOR, p-mTOR, and FAK, were investigated by Western blotting. The results showed decreased expressions of all phosphorylated proteins and FAK in A2058 and A375 cells after treatment (Figure 5).

### 2.5. Bornyl cis-4-Hydroxycinnamate Inhibited MAPK Signaling Pathway-Related Molecules

**Figure 6:**
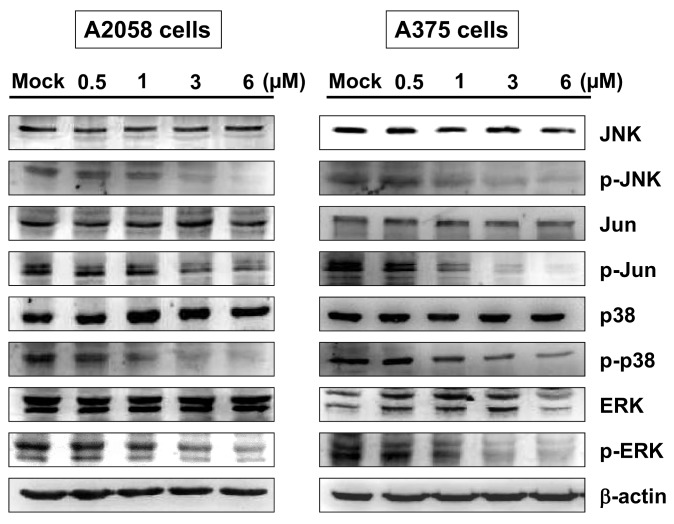
The effects of bornyl *cis*-4-hydroxycinnamate on mitogen-activated protein kinase (MAPK) signaling pathways in A2058 and A375 cells. A2058 and A375 cells were incubated with different concentrations of bornyl *cis*-4-hydroxycinnamate (0, 0.5, 1, 3, 6 µM) for 24 h, and cell lysates were collected for Western blot analysis. JNK, p-JNK, Jun, p-Jun, p38, p-p38, ERK, and p-ERK of MAPKs-related proteins were validated. Bornyl *cis*-4-hydroxycinnamate treatment inhibited p-JNK, p-Jun, p-p38, and p-ERK protein levels in A2058 and A375 cells. β-actin was used as the protein loading control. Mock: cells treated with vehicle control (DMSO).

The mitogen-activated protein kinase (MAPK) pathway has been shown to play a key role in the development of melanoma. The MAPK family consists of several types of protein kinases, including JNK, p38 and ERK. To elucidate the signaling pathways that responded to bornyl *cis*-4-hydroxycinnamate treatment in A2058 and A375 cells, MAPKs signaling pathway-related proteins, including JNK, p-JNK, Jun, p-Jun, p38, p-p38, ERK, and p-ERK, were investigated by Western blot analysis. The results showed decreased expression of all phosphorylated proteins in A2058 and A375 cells after treatment with bornyl *cis*-4-hydroxycinnamate (Figure 6).

### 2.6. Bornyl cis-4-Hydroxycinnamate Inhibited the GRB2 Signaling Pathway

**Figure 7:**
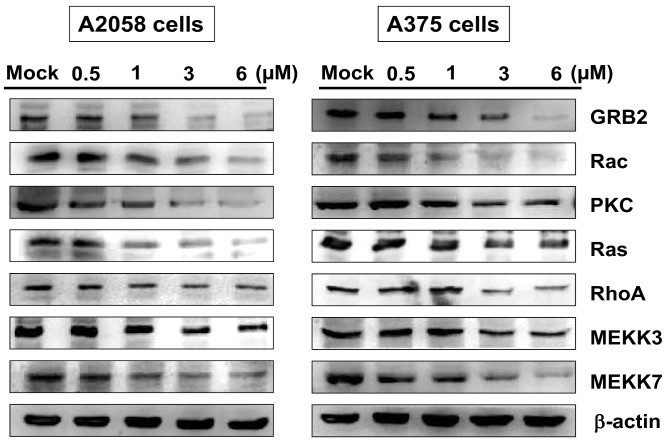
The effects of bornyl *cis*-4-hydroxycinnamate on the GRB2 signaling pathway in A2058 and A375 cells. A2058 and A375 cells were treated with various concentrations of bornyl *cis*-4-hydroxycinnamate (0, 0.5, 1, 3, 6 µM) for 24 h, and cell lysates were collected for Western blot analysis to determine the expression levels of GRB2-related proteins, including GRB2, Rac, PKC, Ras, RhoA, MEKK3, and MEKK7. The protein expression levels were decreased after bornyl *cis*-4-hydroxycinnamate treatment. β-actin was used as the protein loading control. Mock: cells treated with vehicle control (DMSO).

The growth factor receptor-bound protein 2 (GRB2) signaling pathway is known to play a key role in the metastatic process [26]. To determine whether bornyl *cis*-4-hydroxycinnamate-inhibited cell migration is mediated through the GRB2 signaling pathway, proteins involved in the GRB2 signaling pathway, including GRB2, Rac, PKC, Ras, RhoA, MEKK3, and MEKK7, were assessed by Western blotting in A2058 and A375 cells. The levels of GRB2 signal pathway-associated proteins were decreased in A2058 and A375 cells after treatment with bornyl *cis*-4-hydroxycinnamate (Figure 7).

### 2.7. Bornyl cis-4-Hydroxycinnamate Inhibited Epithelial to Mesenchymal Transition (EMT)

**Figure 8:**
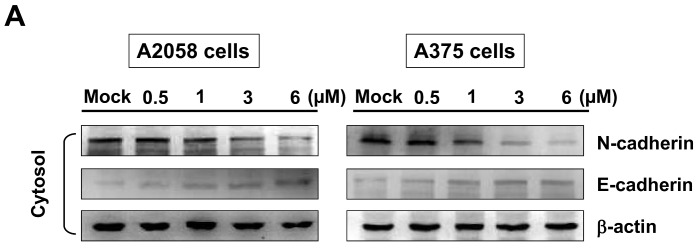

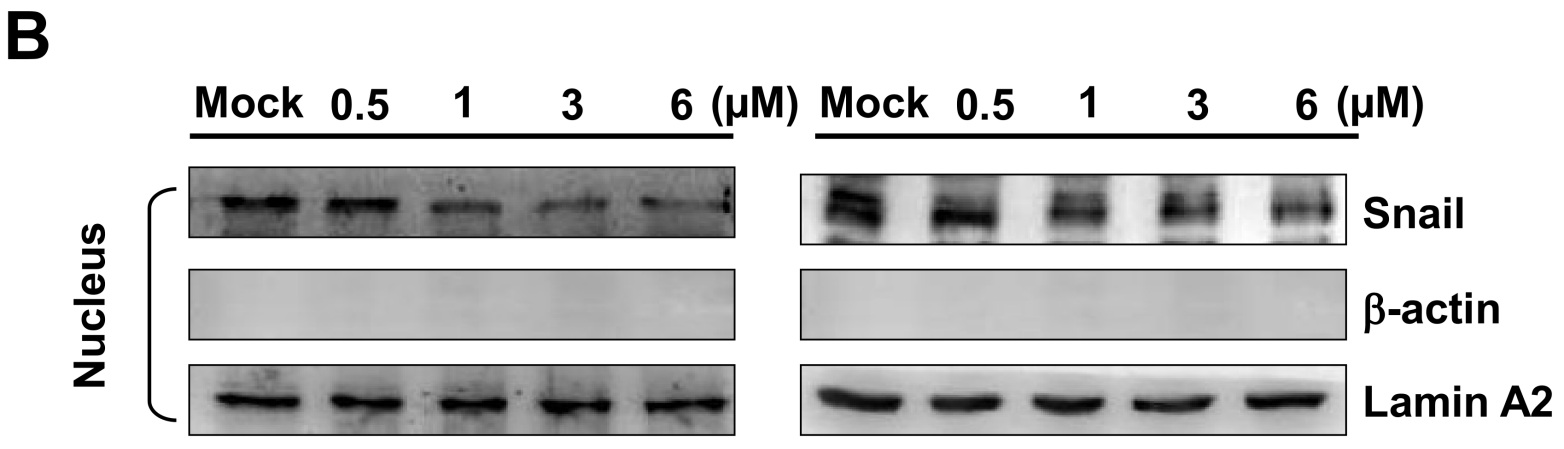
Suppression of epithelial to mesenchymal transition (EMT) by bornyl *cis*-4-hydroxycinnamate in A2058 and A375 cells. A2058 and A375 cells were treated with different concentrations of bornyl *cis*-4-hydroxycinnamate (0, 0.5, 1, 3, 6 µM), and (**A**) cytosol and (**B**) nucleus proteins were collected separately for western blot analysis. EMT-related proteins *N*-cadherin, *E*-cadherin, and Snail were validated. The protein levels of *N*-cadherin and Snail were decreased, while that of *E*-cadherin was increased in A2058 and A375 cells after treatment with bornyl *cis*-4-hydroxycinnamate. β-actin and Lamin A2 were used as the internal controls separately for cytosol and nucleus proteins. Mock: control, DMSO-treated cells.

The epithelial-mesenchymal transition (EMT) is a process by which epithelial cells acquire invasive mesenchymal stem cell-like properties. To determine whether bornyl *cis*-4-hydroxycinnamate inhibited EMT in melanoma cells, we elucidated the expression levels of *E*-cadherin and *N*-cadherin in the cytosol, and Snail in the nucleus. The protein level of *N*-cadherin decreased and that of *E*-cadherin increased in both A2058 and A375 cells after treatment with bornyl *cis*-4-hydroxycinnamate, and the protein level of Snail in the nucleus was also decreased in both A2058 and A375 cells after bornyl *cis*-4-hydroxycinnamate treatment (Figure 8).

## 3. Discussion

A375 is a human amelanotic melanoma cell line derived from a skin biopsy of a 54-year-old female [27]. A2058 is a human melanoma cell line derived from the metastatic site of a lymph node of a 43-year-old male [28]. Our results showed that bornyl *cis*-4-hydroxycinnamate had stronger cell cytotoxic and inhibitory effects on cell migration and invasion in A2058 cells than in A375 cells (Figure 1, Figure 2 and Figure 3), which suggested that bornyl *cis*-4-hydroxycinnamate is more effective in highly-metastatic melanoma cells, and could be a potential therapeutic drug for the treatment of metastatic melanoma.

MMP-2 has been shown to enhance adhesion and migration of melanoma cells via increasing cleavage of fibronectin [29], and MMP-9 overexpression has been found to be associated with tumor melanoma invasion and migration [30]. Previous investigations have shown significant correlations of TIMPs and uPA with the physiological activities of MMP-2 and MMP-9 and their involvement in the invasiveness, metastasis, and prognosis of melanoma [30,31,32]. In addition, uPA is one of the major molecules that contribute to the activation of MMPs, and the expression of uPA has been shown to be associated with the invasive potential of cancers [33]. In the present study, we demonstrated that the signal transduction pathways in bornyl *cis*-4-hydroxycinnamate-treated melanoma cells involve up-regulation of TIMP-1 and TIMP-2 proteins and down-regulation of uPA protein. These proteins may play crucial roles in the inhibition of MMP activity and the anti-invasion properties of bornyl *cis*-4-hydroxycinnamate in melanoma cancer cells. Our results suggested that the inhibitory effects of bornyl *cis*-4-hydroxycinnamate on the activities of MMP-2 and MMP-9 in A2058 and A375 cells may occur via regulation of uPA and TIMP-1/-2.

Focal adhesion kinase (FAK), a cytoplasmic tyrosine kinase, has been shown to play a critical role in the integrin-related signaling pathway, regulating cancer cell migration, invasion, angiogenesis, and epithelial–mesenchymal transition (EMT) in many cell types. Studies have shown that increased expression or activation of FAK is related to cancer metastasis [34,35,36]. We found decreased expressions of FAK, p-PI3K, p-Akt, and p-mTOR in A2058 and A375 cells after bornyl *cis*-4-hydroxycinnamate treatment, which suggested involvement of the FAK/PI3K/Akt/mTOR signaling pathways in the inhibitory effects of bornyl *cis*-4-hydroxycinnamate in malignant melanoma cells. The MAPKs signaling pathway is known to modulate melanin synthesis, and is one of the most commonly investigated molecular-targeted therapies for melanoma [37,38]. Bornyl *cis*-4-hydroxycinnamate treatment inhibited p-JNK, p-Jun, p-p38, and p-ERK protein expression, indicating that the MAPKs signaling pathway is involved in the inhibitory effect of bornyl *cis*-4-hydroxycinnamate in malignant melanoma cells.

GRB2, an adaptor protein involved in cell communication and intracellular signal transduction, has been shown to be important in a number of cellular processes, including actin-based cell motility, angiogenesis and epithelial morphogenesis. These processes are major features in tumor invasion and metastasis [26]. The levels of most of the molecules in the GRB2 signaling pathway were decreased after cells were treated with bornyl *cis*-4-hydroxycinnamate, including GRB2, Rac, PKC, Ras, RhoA, MEKK3, and MEKK7. The findings suggested that the GRB2 signaling pathway is involved in the suppression of cell migration by bornyl *cis*-4-hydroxycinnamate.

EMT is a phenotypic transformation that causes interruption of cell–cell interaction and adhesion, and facilitates cell motility. EMT increases the metastatic potential, and is thought to play important roles in the metastasis and invasion of cancer cells [39,40]. Snail is a transcription factor that triggers EMT, and has been shown to be an important marker for tumor progression and invasion [41,42,43,44]. Disruption of adhesive interactions between melanocytes and keratinocytes mediated by *E*-cadherin [45] is known to coexist with increased *N*-cadherin expression, which facilitates melanoma invasion into and cluster formation within the dermis [46,47,48]. The increased expressions of *E*-cadherin and the decreased expression of *N*-cadherin and Snail in cells treated with bornyl *cis*-4-hydroxycinnamate suggested that bornyl *cis*-4-hydroxycinnamate inhibits cell migration through suppression of the EMT process.

During EMT, extracellular matrix restructuring in tumors typically involves three steps, adhesion, degradation, and migration. The invasive property of the melanoma is also associated with the restructuring of the extracellular matrix. In the current study, we found that in two melanoma cell lines, A2058 and A375, uPA, TIMP-1, MMP-2 and MMP-9 were associated with cell migration and invasion. Most compounds used for cancer therapy inhibit tumor cell progression through one major signaling pathway, and cocktail therapy targets multiple signaling pathways by using more than one compound. Our results showed that bornyl *cis*-4-hydroxycinnamate inhibited human melanoma cell metastasis through the MAPKs, FAK/PI3K/Akt/mTOR and GRB2 signaling pathways. Moreover, bornyl *cis*-4-hydroxycinnamate inhibited EMT in melanoma cells.

## 4. Methods

### 4.1. Materials and Chemical Reagents

The stems of *Piper betle* were collected in Pingtung County, Taiwan in July 2008, which were cultivated by local farmer and bornyl *cis*-4-hydroxycinnamate identified by Chi-I Chang, National Pingtung University of Science and Technology. Rabbit anti-human MMP-2, MMP-9, uPA, TIMP-1, TIMP-2, FAK, PI3K, p-PI3K, Akt, p-Akt, mTOR, p-mTOR, JNK, p-JNK, Jun, p-Jun, p38, p-p38, ERK, and p-ERK were obtained from Cell Signaling Technology (Danvers, MA, USA). GRB2, Rac, PKC, Ras, RhoA, MEKK3, MEKK7, N-cadherin, E-cadherin, Snail, and Lamin A2 antibodies were obtained from Epitomics (Burlingame, CA, USA). Dimethyl sulfoxide (DMSO), protease inhibitor cocktail, and rabbit anti-human β-actin antibodies were purchased from Sigma (St. Louis, MO, USA). PVDF (polyvinylidene difluoride) membranes and goat anti-rabbit and horseradish peroxidase-conjugated IgG were purchased from Millipore (Bellerica, MA, USA). Chemiluminescent horseradish peroxidase (HRP) substrate was purchased from Pierce (Rockford, IL, USA).

### 4.2. Cell Culture

A2058 and A375 cells were purchased from the Food Industry Research and Development Institute (Hsinchu, Taiwan). Cells were cultured in DMEM (Biowest, Nuaillé, France) containing 10% fetal bovine serum (*v*/*v*), sodium pyruvate (1 mM), l-glutamine (4 mM), streptomycin (100 μg/mL), and penicillin (100 U/mL), and maintained in a 37 °C humidified atmosphere incubator with 5% CO_2_.

### 4.3. Cell Viability Assay

For the cell viability assay, cells were seeded at a density of 1 × 10^5^ per well in 24-well culture plates and treated with different concentrations of bornyl *cis*-4-hydroxycinnamate. After 24 h of treatment, the cytotoxicity of bornyl *cis*-4-hydroxycinnamate was determined by thiazolyl blue tetrazolium bromide (MTT) cell proliferation assay (Sigma M2128). Light absorbance values (OD = OD570 − OD620; at wavelengths of 570 and 620 nm) were recorded using an ELISA reader. The results were expressed as a percentage of the control ± SD calculated from *n* = 4 wells per experiment from three independent experiments.

### 4.4. Cell Migration Assay

A2058 and A375 cells were seeded onto a Boyden chamber (Neuro Probe, Cabin John, MD, USA) at a density of 10^4^ cells per well in serum-free media for 24 h, then incubated with different concentrations of bornyl *cis*-4-hydroxycinnamate (0, 1, 3, and 6 µM). After 24 h, the migrated cells on the bottom chamber were fixed by 100% methanol and stained with 5% Giemsa (Merck, Darmstadt, Germany). Cell numbers were observed and counted using a light microscope.

### 4.5. Cell Invasion Assay

For the cell invasion assay, A2058 and A375 cells were suspended in serum-free DMEM, and maintained on the top chamber of transwell inserts coated with Matrigel. DMEM medium containing different concentrations of bornyl *cis*-4-hydroxycinnamate (0, 1, 3, and 6 µM) was added to the lower chamber. After incubation for 48 h, the membranes of the transwell inserts were fixed in neutral formalin. The cells and Matrigel in the upper chamber were removed, followed by Giemsa staining for further analysis of the extent of tumor cell invasion. After Giemsa staining, under a light microscope, the cells that had migrated through the membrane were counted in six randomly-selected fields in order to determine the extent of invasion.

### 4.6. Gelatin Zymography Assay

To determine the activities of MMPs, A2058 and A375 cells were seeded in 12-well plates at a density of 1 × 10^5^ cells/well and incubated in serum-free DMEM with different concentrations of bornyl *cis*-4-hydroxycinnamate (0, 1, 3, 6 µM). After 24 h of incubation, the conditioned media were assayed using gelatin zymography with 8% gelatin gels to determine the activities of MMPs released from A2058 and A375 cells. In brief, the collected conditioned media were electrophoresed in an SDS-PAGE gel containing 0.2% gelatin, and then washed twice in a wash buffer (NaCl (100 mM) and Triton X-100 (2.5%) in Tris-HCl (50 mM) buffer, pH 7.5). The gel was subsequently transferred to a reaction buffer (NaCl (200 mM), NaN3 (0.02%), ZnCl_2_ (1 µM), CaCl_2_ (1 mM), Triton-X 100 (2%), in Tris-HCl (50 mM) buffer, pH 7.5) and underwent enzymatic reaction at 37 °C with shaking overnight. The gel was then stained with Coomassie blue and destained in solution of acetic acid (10% *v*/*v*) and methanol (20% *v*/*v*).

### 4.7. Western Blot Analysis

After stimulation, cells from each well were rinsed twice with ice-cold Phosphate-buffered saline (PBS) and lysed in 100 μL of cell lysis buffer (Tris/HCl (20 mM), pH 7.5, NaCl (125 mM), MgCl_2_ (1 mM), Triton X-100 (1%), NaF (50 mM), β-glycerophosphate (25 mM), Na_3_VO_4_ (100 mM), pepstatin (10 μg/mL), Leupeptin (10 μg/mL), aprotinin (10 μg/mL)). The cell lystates were centrifuged, and supernatants were collected. We used the nuclear extraction kit (Signosis, Santa Clara, CA, USA; catalog number SK-0001) to extract nuclear proteins for detection of Snail. Collected the cells and rinsed with ice-cold PBS and added Buffer I working reagent (1 mL/10 cm dish) to the cells shook at 200 rpm for 10 min. Cells were transferred to a 1.5 mL eppendorf and centrifuged at 12,000 rpm for 5 min at 4 °C. The supernatant was then discarded, and Buffer II working reagents (250 μL/10 cm dish) were added to the cells. Samples were shaken at 200 rpm for two hours, centrifuged sample at 12,000 rpm for 5 min at 4 °C, and the supernatant was collected for Western blot analysis. 25 μg of total cell lysates and nuclear proteins were loaded into wells and separated by 12.5% SDS-PAGE. The protein was then transferred onto PVDF membrane for 1.5 h at 400 mA using Transphor TE 62 (Hoeffer, Holliston, MA, USA). After transfer, the membranes were incubated with a blocking reagent of 5% dehydrated skim milk to block nonspecific protein binding, and then incubated with primary antibodies at 4 °C overnight. Primary anti-human MMP-2, MMP-9, uPA, TIMP-1, TIMP-2, FAK, PI3K, p-PI3K, Akt, p-Akt, mTOR, p-mTOR, JNK, p-JNK, Jun, p-Jun, p38, p-p38, ERK, p-ERK, GRB2, Rac, PKC, Ras, RhoA, MEKK3, MEKK7, *N*-cadherin, *E*-cadherin, snail, and Actin antibodies were used, followed by incubation with secondary antibodies (horseradish peroxidase conjugate goat anti-rabbit, 1:5000 in blocking solution) for 2 h at 4 °C, then detected using chemiluminesence (Pierce Biotechnology, Rockford, IL, USA).

### 4.8. Statistical Analysis

Data of cell viability, cell migration, and cell invasion assays were pooled from three independent experiments. The results were expressed as mean standard error of mean (SEM). Data acquisition was completed and analysis of variance (ANOVA) was carried out using the Tukey-Kramer test, employing GraphPadInStat 3 software (San Diego, CA, USA) to determine between-group statistical significance.

## 5. Conclusions

**Figure 9:**
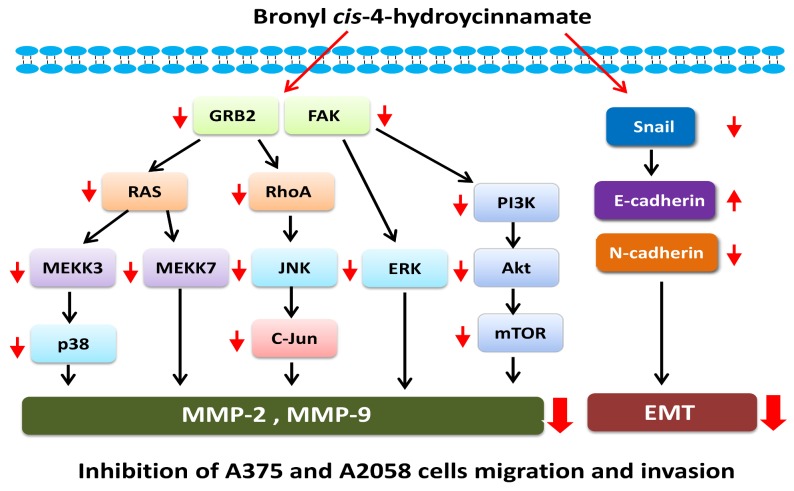
Proposed signaling pathways for bornyl *cis*-4-hydroxycinnamate-mediated inhibition of A2058 and A375 melanoma cell migration and invasion.

Our study results proved that the natural compound bornyl *cis*-4-hydroxycinnamate extracted from *Piper betle* possesses anti-cancer activity consisting of inhibition of cell invasion, cell migration, and the EMT process in A2058 and A375 cells. The results indicated that regulation of MMP-2/-9-related signaling pathways is involved in the process of human melanoma cell metastasis. Bornyl *cis*-4-hydroxycinnamate effectively inhibits cell metastasis through multiple signaling pathways in human melanoma cells (Figure 9). Further in vivo study confirming the potential anti-metastatic effects of bornyl *cis*-4-hydroxycinnamate is needed. The results of the current study suggested that bornyl *cis*-4-hydroxycinnamate may be a potential therapeutic agent for the treatment of human melanoma.

## Supplementary Materials

Supplementary materials can be found at http://www.mdpi.com/1422-0067/19/8/2152/s1.
