# Supplementary material for: Bornyl cis-4-Hydroxycinnamate Suppresses Cell Metastasis of Melanoma through FAK/PI3K/Akt/mTOR and MAPK Signaling Pathways and Inhibition of the Epithelial-to-Mesenchymal Transition
Source: Int J Mol Sci. 2018 Jul 24;19(8):2152. doi: 10.3390/ijms19082152 (PMC6121392; doi:10.3390/ijms19082152)
Supplement: Supplementary file 1 [file ijms-19-02152-s001.pdf]

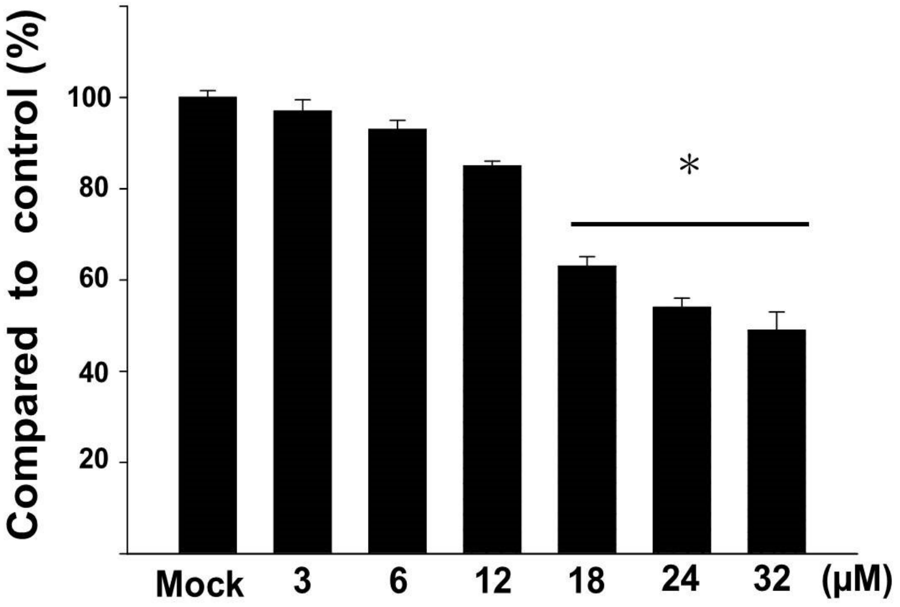

Figure S1. We selected the melanoma cells (B16-F10 cells) for the study and also used different concentration of bornyl cis-4-hydroxycinnamate for the MTT assay. The cell viability assay results were similar to A2058 cells and A375 cells.

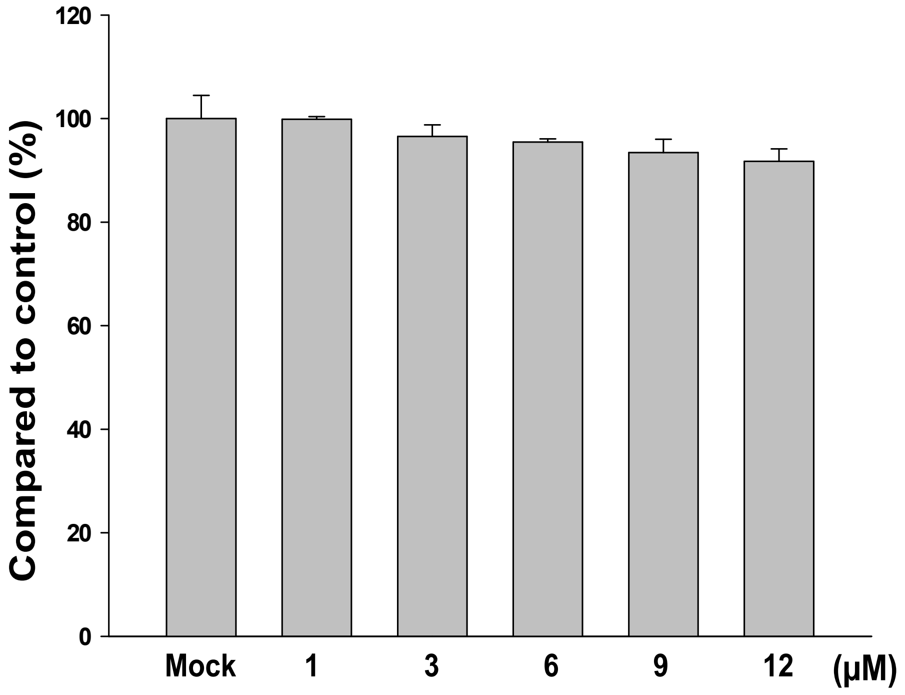

Figure S2. We selected the fibroblast cells (WS-1 cells) for cell cytotoxicity analysis. In the results, we found that bornyl cis-4-hydroxycinnamate was not significantly cytotoxic for WS-1 cells.
